# Preparation and Characterization of New Bioplastics Based on Polybutylene Succinate (PBS)

**DOI:** 10.3390/polym15051212

**Published:** 2023-02-28

**Authors:** Federico Barrino, Harrison De La Rosa-Ramírez, Chiara Schiraldi, Juan López-Martínez, María Dolores Samper

**Affiliations:** 1 Department of Experimental Medicine, University of Campania “Luigi Vanvitelli”, Via L. De Crecchio 7, 80138 Naples, Italy; 2 Institute of Materials Technology (ITM), Polytechnic University of Valencia (UPV), 46022 Alicante, Spain

**Keywords:** bioplastic film, bioplastic, extra virgin oil, coconut oil, food packaging, FT-IR

## Abstract

Sea and environmental pollution due to microplastics are global problems that in recent years have attracted particular interest in the scientific community. The increase in the world population and the consequent consumerism of non-reusable materials are amplifying these problems. In this manuscript, we present novel bioplastics, which are completely biodegradable, for their potential use in food packaging, to replace fossil-fuel-derived plastic films and slow food degradation due to oxidative processes or microbial contamination. In this study, thin films based on polybutylene succinate (PBS) were prepared to reduce pollution, and different percentages by weight (1, 2 and 3 wt%) of extra virgin olive oil (EVO) and coconut oil (CO) were included to improve the chemico-physical properties of the polymer and possibly improve the functionality of the films in terms of prolonged food preservation. Attenuated total reflectance Fourier transform infrared (ATR/FTIR) spectroscopy was used to evaluate the interactions between the polymer and the oil. Furthermore, the mechanical properties and thermal behavior of the films were evaluated as a function of the oil content. A scanning electron microscopy (SEM) micrograph showed the surface morphology and the thickness of the materials. Finally, apple and kiwi were selected for a food-contact test, and the wrapped sliced fruit was monitored and evaluated for 12 days to macroscopically evaluate the oxidative process and/or eventually occurring contamination. The films were shown to reduce the browning of sliced fruit due to oxidation, and no molds were evidenced up to 10/12 days of observation with the addition of PBS, with 3 wt% of EVO achieving the best outcomes.

## 1. Introduction

Food waste and the consequent environmental pollution caused by the dispersion of its packaging material in the environment are certainly among the biggest concerns of these last decades. Especially in Western countries, people dispose of tons of food and its lining every day. Wasting food is not just a question of ethics; it leads to a depletion of the already limited natural resources of our planet. Indeed, much of the food produced for human consumption is wasted along the food supply chain [1]. In Europe, household-generated food waste accounts for more than half of the total food waste, much of which consists of “food that has or had the potential to be consumed” [2]. The large amount of waste produced, such as the coatings that wrap food, has serious environmental consequences: in addition to the waste of resources, it has negative consequences related to greenhouse gas emissions, as well as the pollution of forests and the sea by non-recyclable plastic, thus damaging the ecosystem [3]. At the end of their life cycle, food-packaging plastics become solid waste that pollutes the environment over a long period, as they do not degrade or decompose in natural environments [4]. Furthermore, the production and disposal of synthetic polymers produce CO_2_ and, therefore, contribute to global warming. The reduction in food waste and the consequent environmental pollution is part of the European Green Deal; the EU is committed to reducing food waste and losses by 50% by 2030 [5]. Most of the measures to reduce this problem are based on the voluntary commitment of consumers. Some information campaigns have been conducted to encourage people not to waste and to reuse resources [6], but these approaches may not be sufficient. Since, currently, the most commonly used plastics for food packaging are synthetic plastics, i.e., those made from petroleum, in this study, we focused on the use of renewable and biodegradable resources. The aim of this work is to obtain fully biodegradable bioplastics for use in the food-packaging industry in order to eliminate both CO_2_ emissions during the preparation of the plastics and their accumulation and release into the environment. 

It is reported in the literature that biopolymers degrade easily in the environment and can be classified into three categories: those extracted from biomass, those synthesized from bioderived monomers, and those produced by microorganisms. Furthermore, through methods such as solution casting, electrospinning, and hot pressing or fusion blending and the subsequent extrusion blown film, materials are obtained that can be used for food packaging [7,8]. Polybutylene succinate (PBS) is a versatile semi-crystalline polymer, which is obtained by the simple direct esterification of succinic acid with 1,4-butanediol [9]. Succinate is now obtained in large part from renewable resources by fermentation. Furthermore, 1,4-butandiol biosynythesis was developed recently [10]. Polybutylene succinate is in high demand in the bioplastics industries due to these characteristics; however, compared to conventional plastics, PBS is still very expensive. For this reason, it is often mixed with biopolymers or natural molecules to reduce production costs and to favor different applications [11]. Among natural bioactive agents, oils are increasingly gaining attention in food-packaging applications due to their various biological benefits [12]. In this work, we present a potential combination of this biopolymer with two different types of oil widely used in diverse world macro-areas, specifically Europe and South America. Thin films were prepared based on PBS with extra virgin olive oil (EVO) and coconut oil (CO), at increased concentrations, 1–3 wt%. The low percentages were chosen in order to ensure the mixability and homogeneous distributions of the oils within the PBS matrix during processing and in the final films, and to avoid cost increases for the final material compared with the proposed material. The innovative contribution of this manuscript is the use of active films capable of slowing down the deterioration of food in order to avoid the waste of disposable fruit and vegetables. In fact, fruit and vegetables are too often cut and most are thrown away because the consumer is unable to consume it all. For this reason, the materials obtained have the potential to wrap cut and uneaten fruit in order to be able to preserve it and maintain its integrity over time, thereby to preventing it from being thrown away and wasted.

## 2. Materials and Methods

### 2.1. Materials

#### Polymer and Oils

Two different types of commercial oil were used to obtain the materials: extra virgin olive oil (EVO) and coconut oil (CO). Superior category olive oil obtained directly from olives and only by mechanical means. Produced by Jobellòn, S.L. (Alicante, Spain) and stored at room temperature. High-quality coconut oil made from freshly harvested, sustainably farmed coconuts. Cold-pressed, without any hydrogenation processes. Manufactured by Laboratorios Almond, S.L. (Librilla Murcia, Spain) and stored at room temperature.

Polybutylene succinate (PBS) grade FZ91PM obtained from PTT MCC-Biochem Co., Ltd. (Bangkok, Thailand).

### 2.2. Preparation

#### 2.2.1. Extrusion

The PBS pellets were dried for 12 h at 40 °C in a MDEO model dehumidifier oven from Industrial Marsé (Barcelona, Spain). Subsequently, by mechanical stirring for 5 min, the pellets were premixed with the different percentages by weight of olive oil and coconut oil. To obtain homogeneous mixtures, the different formulations were processed in a co-rotation twin-screw extruder, with length-to-diameter ratio L/D 24, of Dupra, SL (Alicante, Spain). The programmed temperature profiles from the feed hopper to the material outlet nozzle were 110°, 115°, 120° and 125 °C, with a screw speed of 20 rpm. The extruded materials are summarized in Table 1.

#### 2.2.2. Film Manufacturing

**Figure 1:**
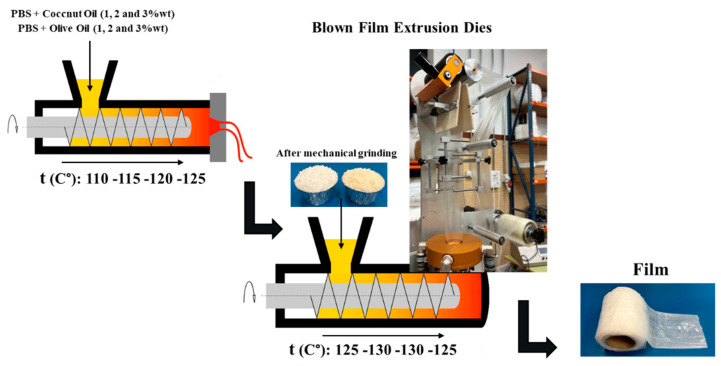
Flowchart for obtaining the film.

After extrusion, the materials were dried at room temperature for 30 min. Successively, they were mechanically grounded with a multifunctional pelletizing unit, MME-01, from ALBI-TECH (Chelm, Poland). After grinding, the new material pellets were inserted into a Microex Blown film extruder from Eurotech Extrusion Machinery Srl (Tradete, Italy) to obtain the films. The programmed temperature profiles from the feed hopper to the material outlet nozzle were 125°, 130°, 130° and 125 °C, with a screw speed of 20 rpm. The flow chart for obtaining the films is shown in Figure 1.

### 2.3. Chemico-Physical and Mechanical Charaterization

#### 2.3.1. Thermal Characterization

The materials’ thermal characterization was carried out in a differential scanning calorimeter (DSC) Mettler-Toledo 821 (Schwerzenbach, Switzerland). To examine the main thermal transitions of neat PBS and PBS formulations, a thermal analysis was programmed in three cycles: (a) initial heating from 25 °C to 180 °C, (b) cooling from 180 °C to −50 °C, and (c) second heating from −50 °C to 350 °C. To evaluate olive oil and coconut oil additives, the thermal analysis was programmed with an initial heating from 30 °C to 90 °C, a cooling from 90 °C to 30 °C and a second heating from 30 °C to 130 °C. Both thermal analyses were conducted at a heating–cooling rate of 10 °C min^−1^, in a nitrogen atmosphere (30 mL min^−1^) using an average sample weight ranging from 6 to 8 mg, placed in standard aluminum crucibles with a volume capacity of 40 μL. The degree of crystallization (*X_c_*%) was calculated with the following Equation (1), where $\Delta H_{m}$ is the melting enthalpy, $\Delta H_{cc}$ is the cold crystallization enthalpy, $\Delta H_{m}^{c}$ is the melting heat associated with pure crystalline PBS, reported to be 110.5 J g^−1^ [13], and $W_{PBS}$ the proportion of PBS in the sample.

(1) $$X_{c}=100\times \left[\frac{\Delta H_{m}-\Delta H_{cc}}{\Delta H_{m}^{c}}\right]\times \frac{1}{W_{PBS}}$$

The samples’ thermal stability was evaluated by thermogravimetric analysis (TGA), conducted in a Linseis TGA PT1000 (Selb, Germany). The dynamic analysis of the decomposition profiles of neat PBS and PBS formulations was conducted with a heating rate of 10 °C min^−1^, from 40 °C to 600 °C, in a nitrogen atmosphere (30 mL min^−1^), using samples with an average weight ranging from 18 to 20 mg placed in standard alumina crucibles (70 μL). The onset degradation temperatures (T5%) were determined at 5% of mass loss, while temperatures of the maximum decomposition rate (Tmax) were calculated from the first derivative of the TGA curves (DTG).

#### 2.3.2. Chemical and Morphological Characterization

The FTIR/ATR Perkin-Elmer infrared spectrometer BX (Madrid, Spain) was used to evaluate the interactions between the oils and the polymer matrix. The oils were analyzed using KBr discs. Each disc was prepared using 90 mg of KBr and 0.3 mg of oil sample. The analysis was performed with Fourier transform infrared transmittance (FTIR) over a wavenumber range of 4000–400 cm^−1^ with a resolution of 4 cm^−1^ (11 scans). The FTIR spectra were processed by the Prestige software (IR solution). The surface morphology of the hybrid materials was studied by scanning electron microscope analysis using AURIGA Zeiss high-resolution field emission equipment (HR-FESEM, JEOL JSM-7000F, Zeiss, Kyoto, Japan).

#### 2.3.3. Mechanical Characterization

The tensile tests were carried out by a universal tensile tester machine ELIB 50 from S.A.E. Iberstest (Madrid, Spain) at room temperature. A minimum of five different samples was tested using a load cell of 5 kN with a loading speed of 10 mm min^−1^, as suggested by ISO 527 standard. Tensile tests were performed in quintupled. Following ISO 527 standards, the films were cut into strips that were 10 mm wide and 100 mm in useful length.

#### 2.3.4. Optical Properties

The light transmission of PBS/EVO and PBS/CO films was determined by UV–Vis spectroscopic analyses carried out on a Cary 100 UV–Vis spectrophotometer by Agilent technologies (Barcelona, Spain). The spectra were recorded in the 700–190-nanometer region. Transparency was calculated by the following Equation (2):

Transparency = A_600_/e (2)

where *A*_600_ is the absorbance at 600 nm and *e* the film thickness (mm).

#### 2.3.5. Static Water Contact-Angle Measurements

The water contact-angle measurements of the film surfaces were conducted at room temperature by the sessile drop method, with an Easy Drop Standard goniometer FM140 (KRÜ SS GmbH, Hamburg, Germany) equipped with a stroboscopic camera and an analyzer program (Drop Shape Analysis SW21; DSA1), using distilled water as contact liquid on the film surface with a micro syringe. After depositing a drop of distilled water (volume between 2 µL–6 µL), an average time of 60 s was allowed to pass before conducting the first measurement, after which four additional measurements were performed at 10-s intervals. This step was performed in triplicate, at random positions on each sample surface.

#### 2.3.6. Food-Contact Test

Fresh fruit with the same batch number, grown in the same way and produced in the same place was used to evaluate the ability of the developed film to preserve it from deterioration through oxidative processes and contamination from molds. The films produced were placed in contact with apples and kiwis cut in half to allow perfect adhesion on the surface. Browning of the fruit surface due to oxidation and mold growth were monitored for up to 12 days at room temperature, without specific atmospheric controls (e.g., vacuum, nitrogen, etc.).

## 3. Results

### 3.1. Thermal Characterization

**Figure 2:**
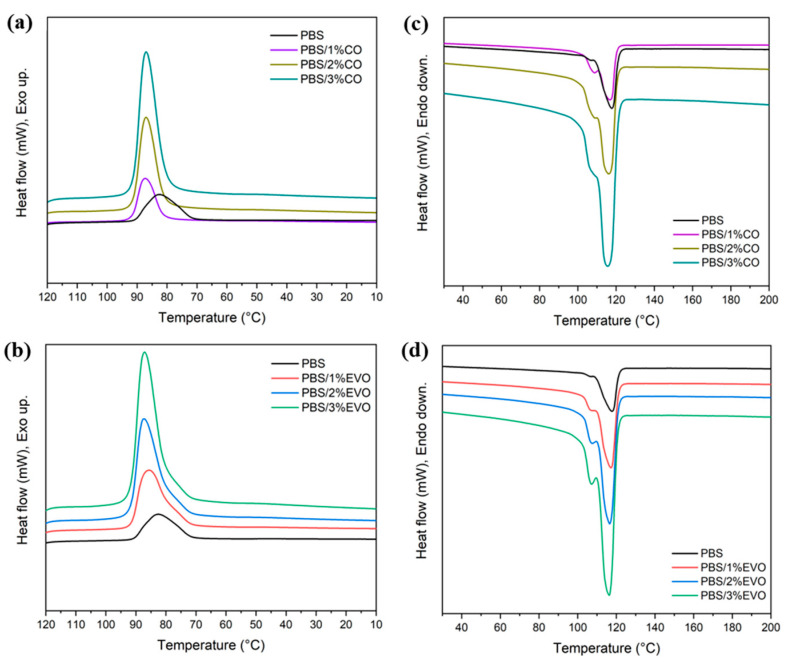
The DSC curves of enthalpy of crystallization: (**a**) PBS and PBS/CO and (**b**) PBS and PBS/EVO. The DSC curves of enthalpy of melting: (**c**) PBS and PBS/CO and (**d**) PBS and PBS/EVO.

The use of differential scanning calorimetry (DSC) allowed us to obtain information on the thermal behavior of the PBS and of the materials obtained by heating or cooling them in a controlled manner. Figure 2 shows the temperatures or temperature ranges in which any transitions occurred (for example, melting or crystallization processes). The DSC analysis measures the heat fluxes that occur in a sample when it is heated/cooled (dynamic conditions) or kept at a constant temperature (isothermal conditions) in a controlled manner. A summary of the main thermal parameters obtained is shown in Table 2.

The cold crystallization temperature (*T_cc_*) increased with the addition of the oils, without significant variations depending on the kind of oil. The exothermic peak located at 82.6 °C, corresponding to the cold crystallization of the pure PBS, was slightly shifted to higher temperatures in all the formulations, reaching 87.9 °C for the PBS/EVO 2 wt% (Figure 2a,b). On the other hand, the melting temperature (*T_m_*) had a decreasing trend. In particular, the endothermic peak located at 117.8 °C corresponding to the melting of the pure PBS was slightly shifted to lower temperatures in all the formulations, reaching values of 114.4 °C for the PBS/CO 3 wt% (Figure 2c,d). Regarding the degree of crystallization, it was observed that the PBS used presented low crystallization, 0.04%, and that the addition of both oils slightly increased the degree of crystallization.

### 3.2. Chemical and Morphological Characterization

**Figure 3:**
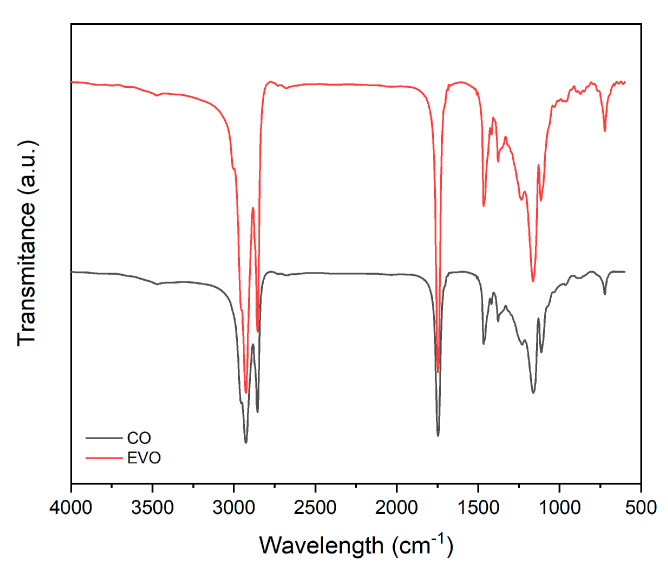
FTIR spectra of (black) coconut oil and (red) olive oil.

Figure 3 shows the FTIR spectra of the olive oil and coconut oil evaluated in the mid-infrared region (4000–650 cm^−1^). Each peak in the FTIR spectra corresponds to the functional groups responsible for infrared (IR) absorption and shows characteristic bands for edible fats and oils [14]. The FTIR spectra of the oils appeared to be very similar; however, some differences were revealed in terms of the band intensity and the exact frequencies at which the maximum absorbance was generated in each oil.

Table 3 reports the FTIR analysis values. The peaks recorded with the corresponding functional groups responsible for the absorption of the IR of the oil samples evaluated are also confirmed in the literature [15].

**Figure 4:**
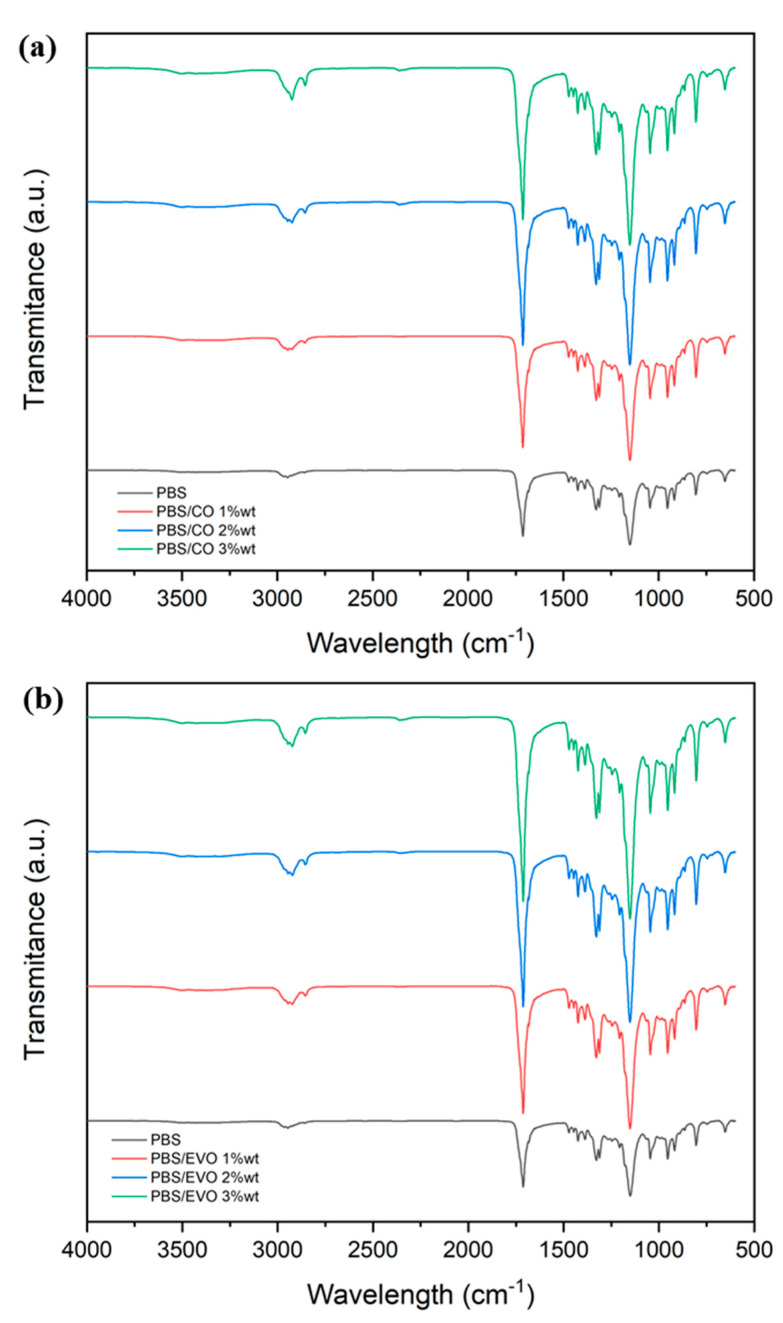
FTIR spectra of: (**a**) PBS and PBS/CO formulations and (**b**) PBS and PBS/EVO formulations.

The attenuated total reflectance–Fourier transform infrared spectroscopy (ATR–FTIR) spectrum of the polybutylene succinate showed an absorption band at 2922 cm^−1^, which was assigned to the stretching of the C–H bond. The intense band recorded at 1711 cm^−1^ characterizes the formation of the ester group and corresponds to the stretching vibration of the C=O carbonyl. Furthermore, the peak at 1328 cm^−1^ was assigned to the elongation vibration of the –COO bond. Finally, the signal at 1160 cm^−1^ is a characteristic of the C–O–C stretching vibration in the repeating unit, –OCH_2_CH_2_ [16]. The interactions of the PBS with the olive oil and the coconut oil were evaluated by ATR–FTIR analysis. In Figure 4a, the spectra of the PBS co-compared with the compositions of PBS + coconut oil are reported, while in Figure 2b, the spectra of the PBS co-compared with the compositions PBS + olive oil are reported. When comparing the spectrum of the PBS with all the others, it is possible to observe some different peaks due to different incorporated oils. When different amounts of oils were added to the materials, the methylene C–H bands that stretched and flexed at 2930 and 2855 cm^−1^ were clearly detectable in all the spectra, and their intensity increased with an increase in the amount of oil. Furthermore, when a high amount of oil was added to the PBS, a different shape of the typical band from 1500 to 800 cm^−1^ was observed, particularly for the peak at 1161 cm^−1^. The intensity of the characteristic bands of the PBS appeared to be strongly influenced by the oil content.

**Figure 5:**
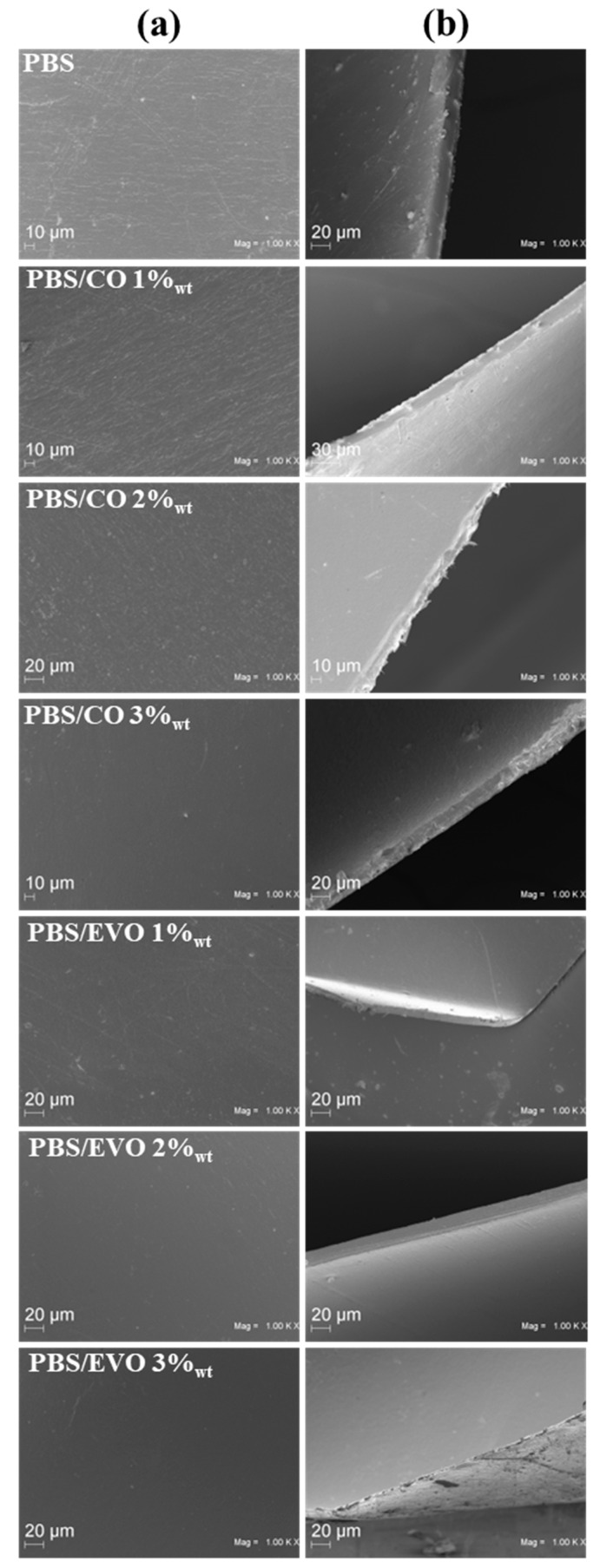
The SEM micrographs of the studied formulations: (**a**) film surfaces and (**b**) film thicknesses.

The films were observed through the SEM and the micrographs of the surfaces obtained are shown in Figure 5a, while the thickness micrographs are shown in Figure 5b. The microstructure showed that both the coconut oil and the olive oil had good compatibility with the polybutylene succinate matrix. In particular, when the maximum percentage of oil was used in the preparation, a homogeneous and smooth surface was observed. In the case of the sample with PBS only, a surface with homogeneous roughness was observed. The same roughness was observed on the surface of the PBS/CO 1 and 2 wt% samples. Thanks to the thickness micrographs, it was possible to calculate the following dimensions (µm): PBS (20.63 ± 0.03); PBS/CO 1 wt% (17.08 ± 0.02); PBS/CO 2 wt% (16.45 ± 0.15); PBS/CO 3 wt% (17.45 ± 0.07); PBS/EVO 1 wt% (8.83 ± 0.05); PBS/EVO 2 wt% (18.31 ± 0.03); and PBS/EVO 3 wt% (2.15 ± 0.08).

### 3.3. Mechanical Charaterization

**Figure 6:**
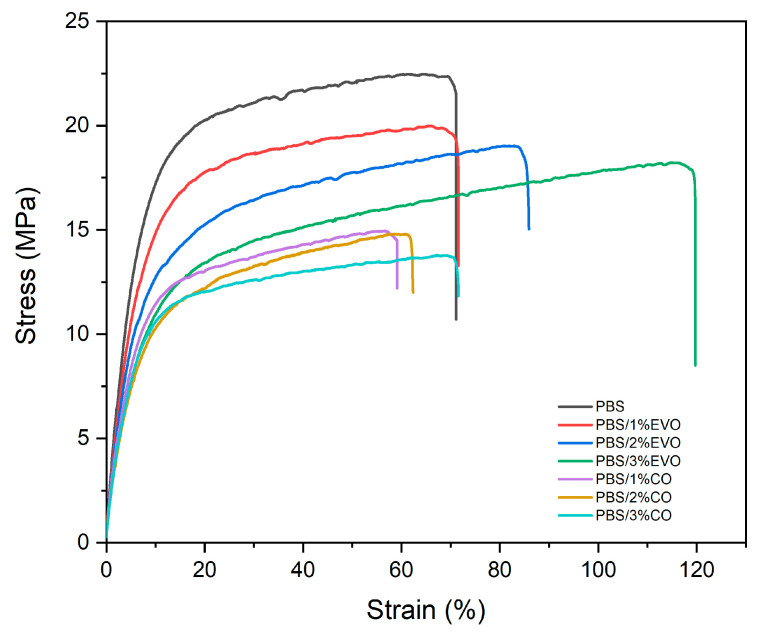
Stress–strain curve of PBS and PBS/formulations.

The evaluation of the tensile properties of the pure PBS and the PBS formulations with the variable oil content was conducted and the results are shown in Figure 6. As shown in this figure, the tensile-strength behavior of the PBS/CO and PBS/EVO blends was similar in both cases. The incorporation of the oils led to a decrease in the tensile-strength values compared to those shown for the pure PBS (22.5 ± 1.29 MPa). It is evident that the addition of coconut oil produced a greater drop in tensile-strength values than the materials formulated with the olive oil. With the same oil content (3 wt%), the tensile-strength values decreased to 13.8 ± 0.80 MPa for the coconut oil and 18.2 ± 1.42 MPa for the olive oil, with a difference of about 4.5 MPa between them. Compared to the PBS, a notable difference was observed between the low strain values and the high stress values for the materials containing the olive oil and the low values of both the strain and the stress for the materials containing the coconut oil. A summary of the main mechanical parameters obtained is reported in Table 4.

### 3.4. Optical Properties

**Figure 7:**
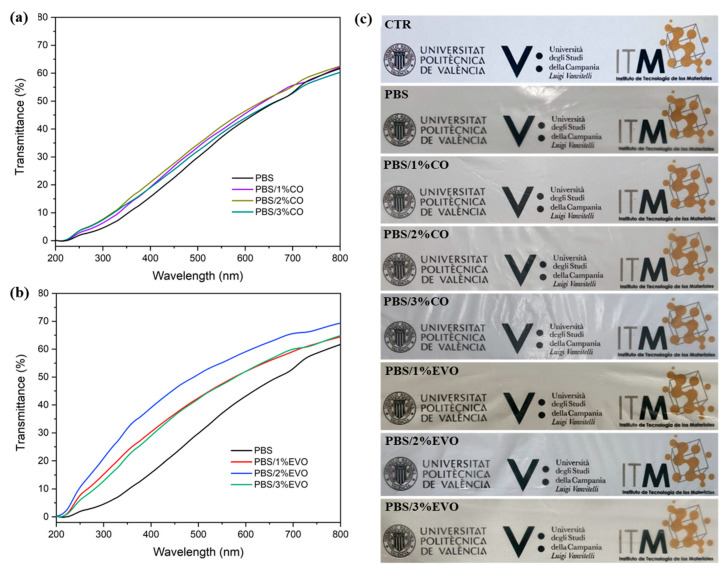
UV–Vis analysis of (**a**) PBS and PBS/CO formulations and (**b**) PBS and PBS/EVO formulations and (**c**) visual appearances of all PBS-based films/formulations.

The absorption spectra of all the materials are shown in Figure 7a,b, while the visual aspects of the samples are shown in Figure 7c. The PBS film showed the highest transmission in the visible-spectrum region (400–700 nm) and the lowest transmission in the UV–A-spectrum region.

Furthermore, the addition of the oils produced opaque materials, and a significant reduction in light transmission in the visible region was observed. Therefore, it seems that the oil reduced the transparency of the films. This resulted in a reduction in the transparency of the materials when increasing the quantity of the oils. The materials showed the following reductions in transparency: PBS (A_600_/mm = 1.13 ± 0.03); PBS/CO 1 wt% (A_600_/mm = 1.19 ± 0.02); PBS/CO 2 wt% (A_600_/mm = 1.54 ± 0.04); PBS/CO 3 wt% (A_600_/mm = 1.77 ± 0.07); PBS/EVO 1 wt% (A_600_/mm = 1.29 ± 0.09); PBS/EVO 2 wt% (A_600_/mm = 1.44 ± 0.09); and PBS/EVO 3 wt% (A_600_/mm = 1.75 ± 0.09).

### 3.5. Water-Absorption Analysis

**Figure 8:**
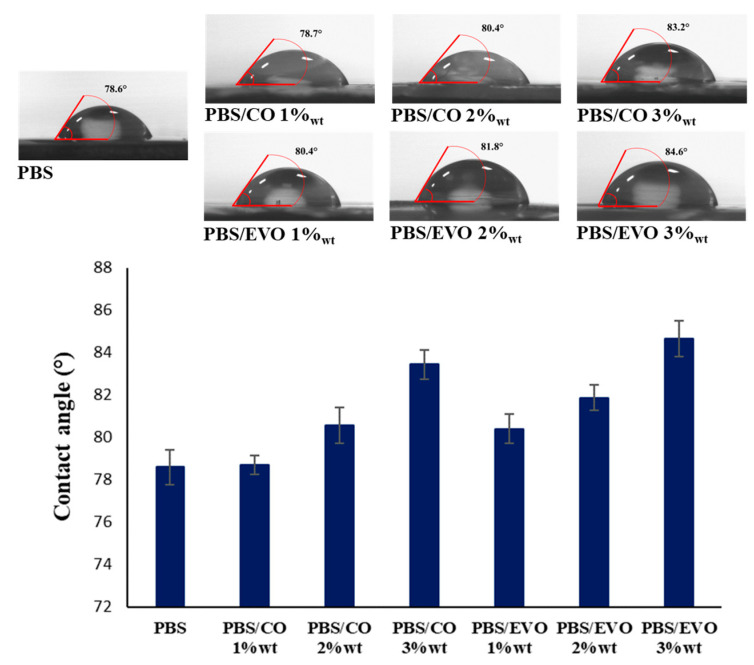
Contact-angle values for PBS and PBS/formulations.

By measuring the contact angle with the water, we investigated whether the addition of oil would have a positive effect on the wettability properties of the PBS. As can be seen in Figure 8, the clean PBS had a contact angle of 78.6. The addition of the oil resulted in an increase in the contact angle of the PBS with the water, showing an increasing trend in values as the amount of oil increased, reaching values of 84.6 for PBS/EVO 3 wt%.

The incorporation of the oil significantly decreased the water-adsorption capacity. The higher the percentage by mass of oil contained, the more hydrophobic the material.

### 3.6. Food-Contact Test

**Figure 9:**
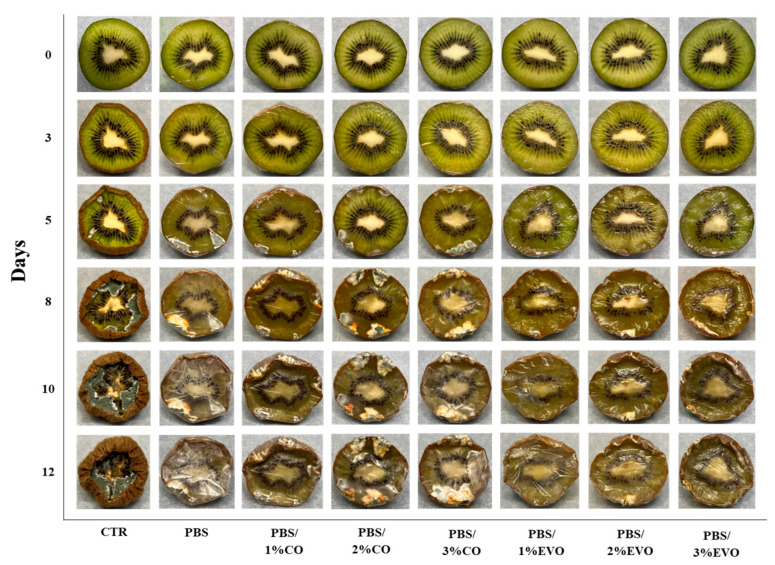
Appearance of fungal growth on sliced kiwi after 12 days of storage with and without PBS and PBS-based films/formulations stored at room temperature.

**Figure 10:**
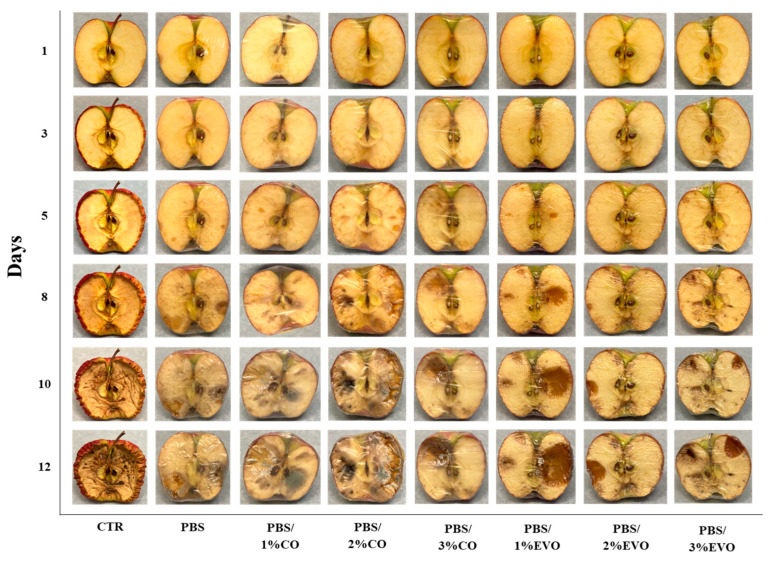
Appearance of fungal growth on sliced apples after 12 days of storage with and without PBS and PBS-based films/formulations stored at room temperature.

The potential of the films developed for use as packaging bioplastics for food preservation was determined by the visual observation of the inhibition of oxidation and molds/fungal growth on the kiwis (Figure 9) and apples (Figure 10). It is possible to observe the pictures of the sliced kiwifruit without the film and with the presence of the films covering the sliced kiwifruit. Compared to time 0 (day 1), all the fruits showed changes in their internal appearance. The activity was monitored for 12 days. After 5 days of incubation, in the control kiwifruit fruits covered with PBS and PBS/CO films, it was possible to observe a phenomenon of fungal growth, which was not present in the case of the PBS/EVO films. After 8 days of incubation, the fruits coated with PBS/EVO films also showed slight fungal growth. In addition, the control and the fruits covered with PBS and PBS/CO films showed exponential fungal growth until the end of the experiment. After 12 days of incubation, the control fruit and the PBS-coated kiwi were almost completely covered by bacteria and fungi. Instead, the fruits coated with polymeric film loaded with olive oil underwent less variation than those submitted to the other treatments. The presence of the olive oil in the films reduced the fungal growth, confirming their potential application in the food-packaging sector.

## 4. Discussion

After proceeding with the extrusion of all the materials at high temperatures, the use of differential scanning calorimetry (DSC) allowed us to obtain information on the thermal behavior of the PBS alone and of those with the addition of olive or coconut oil [17]. It was observed that the melt crystallization temperature (*T_mc_*) increased. This behavior could be ascribed to the anticipation of the melt crystallization process caused by the oils’ incorporation. The oils may have acted as nucleating agents encouraging crystal formation [18,19], increasing the degree of crystallization. On the other hand, the endothermic peak was shifted to lower temperatures as the percentage of the olive and coconut oils increased. As previously reported by Peñas et al. [20], PBS has a symmetrical chemical structure, which facilitates the formation of a highly ordered crystalline structure, which in turn provides a high melting point. 

By measuring the contact angle with the water, we investigated whether the addition of the CO and EVO would have a positive effect on the wettability properties of the PBS, as was previously reported in a study on PLA water-contact angles [21]. Clean PBS has a smaller contact angle than all other formulations. Indeed, the addition of the oils led to an increase in the contact angle of the PBS with the water. This may be ascribed to the oils’ compositions, which include triglycerides, i.e., non-polar hydrocarbons consisting of long chains of fatty acids with COOH functional groups [22]. However, as can be seen from the SEM images, the addition of the oil led to smoother and more uniform hydrophobic surfaces as the percentage by weight contained in the formulations increased. 

The interactions between the polymer and the oils in the hybrid materials were confirmed by the FTIR–ATR spectra. Comparing Figure 3 and Figure 4, it is possible to observe some different peaks due to the different oils incorporated in the hybrid materials. When different amounts of oils were added to the films, the bands of CH_2_ that stretched and flexed at 2930–2855 cm^−1^ were clearly detectable in all the spectra, and their intensity increased as the amount of oil incorporated increased [23,24]. This phenomenon could be said to demonstrate a real interaction between the polymer and the oils. Furthermore, as the percentage by weight of oil in the polymer increased, the intensity of the band increased to 1765 cm^−1^ of the foreign group, C=O [25].

By comparing the mechanical resistance of the clean polymer and the materials obtained, different behaviors were observed between the formulations with CO and EVO. The PBS/EVO 3 wt% formulation was found to be the best because, although it showed a decrement in tensile strength (MPa), it also shows a balance with the increment in elongation at break (%). The reduction in tensile strength was attributed to the initiation of phase separation in the blends containing polymer and oils due to a saturation effect, which weakened the polymer by generating stress concentrations [26]. However, the emergence of a plasticizing effect could probably be discussed in the exclusive case of PBS/EVO 3 wt%, in which a considerable decrease in the tensile modulus and a strong increase in the elongation at break were observed with respect to both the tensile strength and elongation values at break obtained for the pure PBS. Ferri et al. reported a similar plasticizing effect of an epoxy fatty-acid ester an increase in the elongation at break, and a reduction in the elastic modulus and tensile strength in their formulations [27]. 

The small differences in the optical properties and transparency of the polymer and hybrid materials obtained could be very interesting, for example, for use in fruit-film applications, as a slight reduction in UV rays through the films is likely to reduce unwanted photooxidation reactions in plant pigments without hiding the surface of the fruit [28], thus increasing the interest of potential buyers. 

Finally, the potential application of films in the food-packaging sector can be confirmed from the visual analysis of the food-contact test. This is because the kiwi fruits coated with the polymeric film loaded with the highest quantities of oils, particularly EVO, underwent a minor variation compared to other treatments, especially regarding the kiwi samples coated with the pure PBS [29]. Therefore, the presence of the extra virgin olive oil in the films significantly reduced the fungal and bacterial growth.

This preliminary characterization allowed us to suggest a potential use of PBS enriched with oils in food-packaging applications. Further studies on water-vapor permeability (WVTR), oxygen permeability (OTR), and, eventually, antimicrobial activities should be conducted to further investigate the use of these developed materials in specific applications.

## 5. Conclusions

The mixing of PBS and commercial extra virgin oils resulted in film-forming materials that were successfully obtained by means of extrusion at high temperatures and subsequent blown film extrusions. The films were fully characterized from a chemico-physical and mechanical viewpoint. Furthermore, they proved able to slow the oxidation of the sliced fresh fruits. All these results may suggest that PBS-enriched films as potentially good candidates for food-packaging purposes, in order to replace fossil-fuel-derived plastics. In particular, these new forms of packaging may prevent the waste of fruit and vegetables, which, once cut, must be thrown away if not consumed or refrigerated. Based on the results of the food-contact test, the fruit can be kept for several days without undergoing significant alterations at room temperature.

## Figures and Tables

**Table 1:** Composition and labeling of prepared systems.

Label	Composition		
	Polymer (wt%)	Olive Oil EVO (wt%)	Coconut Oil (wt%)
PBS	100	---	---
PBS/CO 1 wt%	99	---	1
PBS/CO 2 wt%	98	---	2
PBS/CO 3 wt%	97	---	3
PBS/EVO 1 wt%	99	1	---
PBS/EVO 2 wt%	98	2	---
PBS/EVO 3 wt%	97	3	---

**Table 2:** Thermal properties of the studied formulations.

Samples	DSC (at a Stream Rate of 10 °C min^−1^)				
	*T_cc_* (°C) ^1^	Δ*H_cc_* (Jg^−1^) ^2^	*T_m_* (°C) ^3^	Δ*H_m_* (Jg^−1^) ^4^	*X_c_* (%) ^5^
PBS	82.6	69.2	117.8	74.1	0.04
PBS/CO 1 wt%	87.2	66.9	116.8	74.9	0.07
PBS/CO 2 wt%	86.7	64.1	114.9	68.1	0.04
PBS/CO 3 wt%	86.7	66.3	114.4	71.6	0.05
PBS/EVO 1 wt%	86.9	70.8	116.7	76.2	0.05
PBS/EVO 2 wt%	87.9	63.3	116.3	68.8	0.05
PBS/EVO 3 wt%	86.8	66.7	115.2	72.1	0.05

^1^ Melt crystallization. ^2^ Melt crystallization enthalpy. ^3^ Melting peak temperature. ^4^ Melting enthalpy. ^5^ Degree of crystallization.

**Table 3:** Functional groups and mode of vibration from FTIR spectra of the evaluated oils.

Peak (cm^−1^)	Assignment of Bonds	Mode of Vibration
3006	=C–H	Stretching
2930	=C–H (CH_2_)	Stretching (asymmetrical)
2855	=C–H (CH_2_)	Stretching (symmetrical)
1747	C=O	Stretching
1466	C–H (CH_2_)	Bending (scissoring)
1415	=C–H	Bending (rocking)
1377	C–H (CH_2_)	Bending (symmetrical)
1161	CH_2_	Bending
1113, 1095, 1028	C–O	Stretching
965	CH=CH	Bending out of plane
858	=CH_2_	Wagging
722	CH=CH	Bending out of plane

**Table 4:** Tensile strength, tensile modulus, and elongation at break of PBS and PBS/formulations.

Sample	Tensile Strength (Mpa)	Tensile Modulus (Mpa)	Elongation at Break (%)
PBS	22.5 ± 1.29	361.5 ± 1.91	71.1 ± 2.22
PBS/CO 1 wt%	15.0 ± 2.06	291.6 ± 10.5	59.2 ± 2.80
PBS/CO 2 wt%	14.8 ± 1.47	226.3 ± 9.44	62.4 ± 3.11
PBS/CO 3 wt%	13.8 ± 0.80	249.1 ± 9.99	71.6 ± 2.21
PBS/EVO 1 wt%	20.2 ± 1.71	336.4 ± 2.76	71.9 ± 2.95
PBS/EVO 2 wt%	19.0 ± 2.27	300.4 ± 4.18	85.9 ± 2.28
PBS/EVO 3 wt%	18.2 ± 1.42	244.8 ± 8.60	119.8 ± 3.21

## Data Availability

Not applicable.
